# Molecular Mechanisms of the Melatonin Receptor Pathway Linking Circadian Rhythm to Type 2 Diabetes Mellitus

**DOI:** 10.3390/nu15061406

**Published:** 2023-03-15

**Authors:** An-Yu Xia, Hui Zhu, Zhi-Jia Zhao, Hong-Yi Liu, Peng-Hao Wang, Lin-Dan Ji, Jin Xu

**Affiliations:** 1 Department of Clinical Medicine, School of Medicine, Ningbo University, Ningbo 315211, China; 2 Department of Internal Medicine, School of Medicine, Ningbo University, Ningbo 315211, China; 3 Department of Preventive Medicine, School of Medicine, Ningbo University, Ningbo 315211, China; 4 Department of Biochemistry, School of Medicine, Ningbo University, Ningbo 315211, China; 5 Zhejiang Key Laboratory of Pathophysiology, School of Medicine, Ningbo University, Ningbo 315211, China

**Keywords:** circadian rhythm, melatonin receptor, T2DM, molecular mechanism

## Abstract

Night-shift work and sleep disorders are associated with type 2 diabetes (T2DM), and circadian rhythm disruption is intrinsically involved. Studies have identified several signaling pathways that separately link two melatonin receptors (MT_1_ and MT_2_) to insulin secretion and T2DM occurrence, but a comprehensive explanation of the molecular mechanism to elucidate the association between these receptors to T2DM, reasonably and precisely, has been lacking. This review thoroughly explicates the signaling system, which consists of four important pathways, linking melatonin receptors MT_1_ or MT_2_ to insulin secretion. Then, the association of the circadian rhythm with *MTNR1B* transcription is extensively expounded. Finally, a concrete molecular and evolutionary mechanism underlying the macroscopic association between the circadian rhythm and T2DM is established. This review provides new insights into the pathology, treatment, and prevention of T2DM.

## 1. Introduction

Circadian rhythm, as the most important inherent biological rhythm, is identified in virtually all eukaryotic organisms. Through the autonomic nervous system and complex hormonal fluctuations, it is deeply involved in the synchronization of physiological functions and daily activities with environmental rhythms [1,2]. If desynchronization occurs, as commonly observed in persons with sleep problems, it will elevate multiple health risks, especially type 2 diabetes, and related cardiovascular complications [3,4,5].

Sleep problems, especially those involving sleep deprivation and circadian rhythm disruption, have been extensively studied. It was estimated that sleep time has markedly reduced, from 8.5 h per day in 2012 to 7.1 h per day in 2021 in China (http://www.zgsmyjh.org, accessed on 11 November 2022), and similar declines have been reported worldwide [6]. As mentioned above, disturbance of the circadian rhythm significantly elevates the risk of type 2 diabetes mellitus (T2DM) [7], which accounts for 90% of diabetes patients [8]. As a rising health burden, the number of adults living with diabetes was estimated to reach 537 million in 2021, corresponding to 10.5% of the world population. Although the epidemiological association between circadian rhythm and T2DM has been reported [9,10], the underlying mechanisms are complex and remain unclear. Melatonin and its receptors serve as intermediaries in synchronizing human endogenous circadian rhythm with environmental rhythms [11]. When people experience circadian rhythm disruption, the central circadian timing system sends incorrect signals that interfere with melatonin release, resulting in the disruption of glucose metabolism, even leading in some cases to T2DM [12]. Numerous studies have explored the intermediary role of the melatonin system, specifically, related signaling pathways, in T2DM. However, few reports have presented a comprehensive summary of the melatonin signaling pathways involved in T2DM.

Thus far, three types of melatonin receptors have been discovered. Among them, MT_1_ and MT_2_ are widely distributed in almost all mammals, whereas MT_3_ is found only in a few animal species. Genome-wide association studies (GWAS) have led to the identification of a strong association between MT_2_-encoding gene (melatonin receptor 1B, *MTNR1B*) polymorphisms and an altered risk of T2DM. Importantly, our previous work revealed that *MTNR1B* is subjected to strong natural selection due to sunshine exposure in human populations worldwide [13]. The combination of two pieces of evidence has suggested the potential molecular and evolutionary mechanisms of *MTNR1B* action in T2DM, and their regulation through circadian rhythm regulation. Therefore, this review systemically explicates the melatonin signaling pathways related to T2DM, and a reasonable evolutionary mechanism is explained.

## 2. Melatonin and Its Receptors

Blood melatonin levels are regulated by sunshine exposure; that is, melatonin is released by the pineal gland at night, but its secretion is inhibited in the daytime. Therefore, melatonin release shows a close relationship with sunshine duration [14]. This phenomenon has been verified in experiments with artificial light exposure [15].

As a receptor for light, the retina transforms optical signals into bioelectrical signals, which are then delivered through the optic nerve to the suprachiasmatic nucleus (SCN), the central pacemaker of the circadian timing system. The SCN is critical for regulating the signals transmitted via sympathetic nerves from the paraventricular nucleus (PVN) to the pineal gland, which controls the secretion of melatonin [11]. More specifically, the PVN first communicates with the higher thoracic segments of the intermediolateral spinal column (ISC), from which the sympathetic nerve extends to the superior cervical ganglion, and then, signals are transferred to postsynaptic fibers. After that, the sympathetic postsynaptic nerve reaches the pineal gland, controlling the synthesis of melatonin. From a molecular perspective, melatonin synthesis involves the conversion of tryptophan to melatonin. Specifically, tryptophan is first hydrolyzed by a tryptophan hydroxylase to form 5-hydroxytryptophan, which is further converted into serotonin, and then, serotonin is acetylated by arylalkylamine *N*-acetyltransferase (AANAT) to yield *N*-acetylserotonin. Finally, under the catalysis of acetylserotonin O-methyltransferase, *N*-acetylserotonin is methylated to become *N*-acetyl-5-methoxytryptamine, namely melatonin [16].

The secretion of melatonin is regulated by different neurotransmitters and nerve conduction systems. As exemplified by the effects of SCN’s regulation on the PVN, astrocytes inside the SCN are first activated to release Glu, which interacts with the NMDA receptor 2 *C*-terminal (NR2C) in the dark. Next, the activated NR2C promotes neuronal secretion of gamma-aminobutyric acid (GABA). Notably, these neurons are active only in the dark; therefore, GABA cannot be secreted in large amounts during the day. Additionally, GABA has been reported to exert an inhibitory effect on the transmission of sympathetic signals [17], which results in PVN inhibition by the SCN [18]. In other words, PVN activity is inhibited in a dark environment, since a large amount of GABA is released from the SCN at night, but in the daytime, GABA levels are reduced and its inhibitory effect is negligible, allowing PVN to be activated.

**Figure 1:**
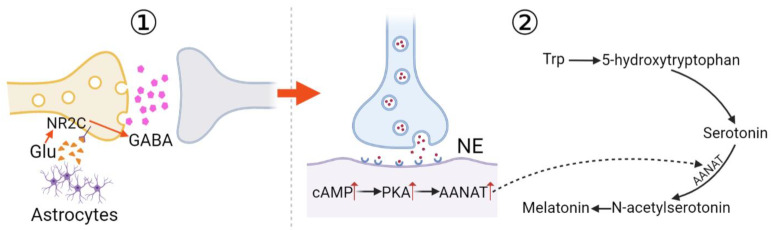
The regulation of melatonin synthesis in the dark. In the dark, melatonin synthesis in the pineal gland is regulated through successively transmitted nerve impulses. ① Astrocytes in the SCN are activated and release Glu, which interacts with NR2C, inducing the release of GABA. ② The sympathetic postsynaptic nerves release norepinephrine to activate AANAT, which promotes the synthesis of melatonin. AANAT: arylalkylamine-*N*-acetyltransferase; GABA: γ-aminobutyric acid; Glu: glutamate; NE: norepinephrine; NR2C: NMDA receptor 2 *C*-terminal; Trp: tryptophan. Both the red arrows in graph ① and black arrows in graph ② show the directions of signaling pathways, while the red upward arrows in graph ② represent concentration increases of each molecules.

Through complex interactions involving nerve impulse transmission, norepinephrine release from the sympathetic postsynaptic fibers, stretching from the superior cervical ganglion, is increased. Subsequently, the released norepinephrine interacts with the β_1_/α_1b_ adrenergic receptors on the pineal cells, increasing intracellular cAMP concentration, and then, activating protein kinase A (PKA). Next, the activated PKA activates AANAT. Hence, in a dark environment, the transformation of tryptophan into melatonin is activated by norepinephrine release (Figure 1). In contrast, during the day, low SCN neuron activity results in low GABA production, relieving the inhibitory effect on the PVN. Therefore, the postsynaptic nerve in the superior cervical ganglion remains in a resting state, and pineal melatonin synthesis is suppressed.

## 3. Melatonin Signaling Pathways in T2DM

As typical G protein-coupled receptors (GPCRs), MT_1_ and MT_2_ distribute more widely than MT_3_. Additionally, compared to either MT_1_ or MT_2_, MT_3_ shows lower affinity for melatonin [19].Therefore, human studies tend to focus on the effects of MT_1_ and MT_2_ [20,21]. In adult human islet cells, MT_2_ has been found to be expressed at a level several fold higher than MT_1_ in both α and β-cells, while MT_2_ expression has been shown to be similar between these two types of cells [22]. Generally, two circumstances trigger the concrete pathways that mediate the influence of MT_1_ and MT_2_ on blood glucose [20]. When MT_1_ and MT_2_ are expressed independently, usually in the plasma membranes as monomeric receptors, the G_i_ protein is recruited and conveys inhibitory signals to adenylate cyclase. However, when MT_1_ and MT_2_ form heterodimers, the IP_3_ pathway is activated, in which the G_q_ protein is initially recruited and transduces signals to phospholipase C (PLC). The association of these melatonin signaling pathways with T2DM is elucidated in detail below.

### 3.1. cAMP Pathway

**Figure 2:**
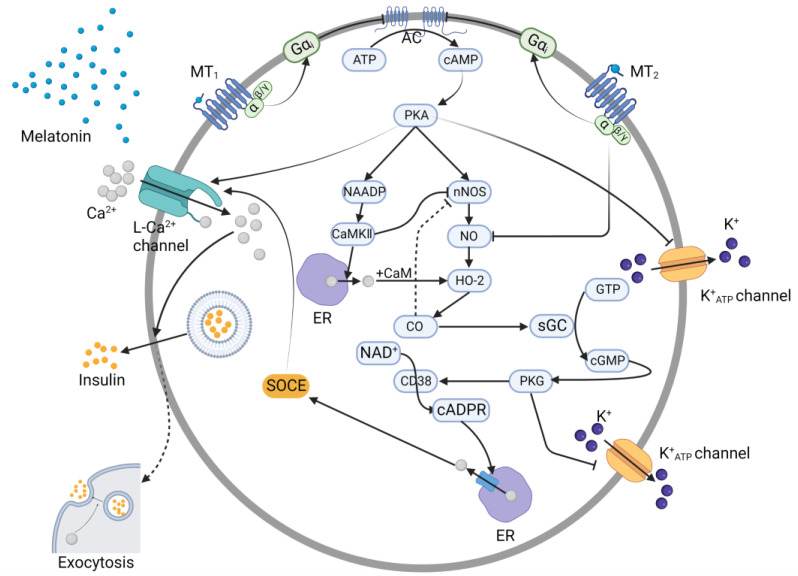
The regulation of melatonin receptors (MT_1_/MT_2_) on the secretion of insulin mediated via cAMP and cGMP pathways. Through MT_1_ and MT_2_ activation, the catalytic activity of AC is downregulated, leading to reduced activation of PKA. Then, PKA is inactivated in one of three pathways, the cAMP pathway, the cGMP pathway, and the K^+^_ATP_ channel, to regulate insulin secretion in combination. AC: adenylate cyclase; ATP: adenosine triphosphate; cADPR: cyclic ADP-ribose; cAMP: cyclic AMP; CaMKII: Ca^2+^/calmodulin-dependent protein kinase II; cGMP: cyclic GMP; CO: carbon monoxide; ER: endoplasmic reticulum; Gα_i_: the α subunit of the G protein; GTP: guanosine triphosphate; HO-2: heme oxygenase 2; nNOS: neuronal nitric oxide synthase; NAD^+^: nicotinamide adenine dinucleotide; NAADP: nicotinic acid adenine dinucleotide phosphate; NO: nitric oxide; PKA: protein kinase A; PKG: protein kinase G; sGC: soluble guanylyl cyclase; and SOCE: store-operated Ca^2+^ entry.

Through a GPCR, the binding of melatonin induces conformational changes in both the monomeric receptors MT_1_ and MT_2_, thereby recruiting the trimeric G_i_ protein to them. After the G_i_ protein binds to either MT_1_ or MT_2_, the α subunit dissociates from the G_i_ protein, and GDP is converted to GTP. The free Gα_i_ subunit moves to adenylate cyclase, inhibiting its ability to transform ATP into cAMP. As a second messenger, cAMP activates PKA, and then, PKA phosphorylates Ser^1928^ in the α_1_ 1.2 subunit, and Ser^478^ and Ser^479^ in the β_2a_ subunit of the L-type Ca^2+^ channel [23,24]. With the phosphorylation of Ca^2+^ channels, the rate of Ca^2+^ influx increases, which facilitates the fusion of insulin-containing secretory vesicles with the plasma membrane [25]. However, because of the inhibitory effect of the Gα_i_ subunit, the binding of melatonin results in low levels of insulin secretion through the cAMP pathway (Figure 2).

### 3.2. cGMP Pathway

PKA acts not only directly on L-type Ca^2+^ channels but also indirectly to regulate Ca^2+^ channel activity. It induces the production of nicotinic adenine dinucleotide phosphate (NAADP) [26], the initial Ca^2+^ signal second messenger. NAADP phosphorylates Ca^2+^/calmodulin-dependent protein kinase II (CaMKII) at Thr^286^, and then, Ca^2+^ is mobilized, moving from the endoplasmic reticulum (ER) via the action of phosphorylated CaMKII [27]. Hence, the intracellular Ca^2+^ concentration increases, and the majority of the Ca^2+^ in the cytosol is combined with calmodulin (CaM) to form the Ca^2+^–CaM complex [27], a precursor in the activation of HO-2.

Meanwhile, PKA enhances the activity of nNOS by phosphorylating it at Ser^1412^ [28], with CaMKII phosphorylating it at Ser^847^, to exert an inhibitory effect [29]. The comprehensive effect is positive, which can be deduced from the end product of the pathway. Then, NO is synthesized, which in turn activates HO-2 via the action of the Ca^2+^-CaM complex. HO-2 then produces CO [27], which is considered to be the activator of soluble guanylate cyclase (sGC). When it is in excess, CO can exert an additional inhibitory effect on nNOS. Activated sGC also convert GTPs to cGMP, which in turn activates PKG. PKG targets the CD38 on endosomes. The activation of CD38 leads to the transfer of NAD^+^ to cADPR, another important Ca^2+^ signal second messenger. cADPR then mediates Ca^2+^ release from the ER, a process called store-operated Ca^2+^ entry (SOCE). Ca^2+^ continues to induce transmembrane Ca^2+^ channel opening, resulting in Ca^2+^ influx and resultant insulin secretion [30]. Noticeably, melatonin-bound MT_2_ was found to exert an additional negative effect on the synthesis of NO [31], suggesting that the MT_2_ pathway exerts an additional inhibitory impact on insulin synthesis.

Furthermore, PKG together with PKA exerts an inhibitory effect on K^+^_ATP_ channels. The K^+^_ATP_ channel enables K^+^ efflux, opening when the intracellular ATP concentration decreases, accelerating membrane repolarization. The duration of the action potential decreases; therefore, the activity and the functional duration of the voltage-dependent Ca^2+^ channel (VDCC) is decreased, and then, the Ca^2+^ influx is decreased. Since both PKA and PKG inhibit the activity of K^+^_ATP_ channels, they simultaneously increase Ca^2+^ influx and insulin secretion [31].

Eventually, as mentioned above, intracellular Ca^2+^ facilitates the fusion of the insulin-containing secretory vesicles with the plasma membrane, thereby transporting insulin out of the cells (Figure 2). However, due to the inhibitory cascade activated by adenylate cyclase, the whole process originating from melatonin production, exerts a negative impact on insulin secretion. That is, the interaction between melatonin and its receptors decreases the amount of insulin secreted.

### 3.3. The IP_3_ Pathway

After receptors MT_1_ and MT_2_ on β-cells simultaneously bind melatonin, the Gα_q_ subunit is activated, allowing it to leave the trimeric G_q_ protein complex and bind phospholipase C (PLC) [31]. However, whether the impact of the Gα_q_ subunit on PLC is positive remains unclear. In light of the inhibitory effect of melatonin on gonadotropin-releasing hormone (GnRH)-induced Ca^2+^ oscillation detected by Zemkova [32,33], it has been speculated that melatonin depresses intracellular Ca^2+^ store release, which is activated by IP_3_. Therefore, the impact of the Gα_q_ subunit on PLC is thought to be negative. However, Andreas et al. [34] showed the opposite effect of melatonin on IP_3_. The difference in outcome may be attributable to the different materials used for these experiments. In Zemkova’s research, the anterior pituitary gland was the subject, while Andreas et al. studied rat insulinoma (INS1) cells.

**Figure 3:**
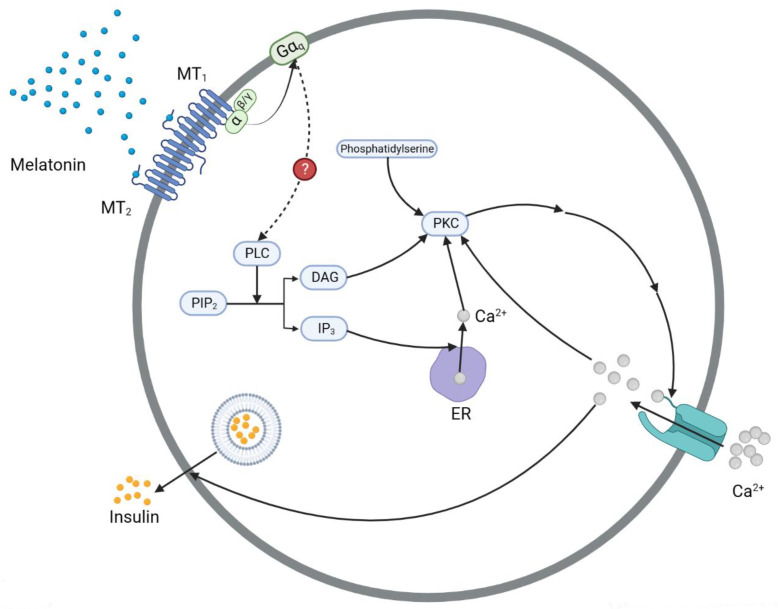
The regulatory effect of melatonin receptors (MT_1_, MT_2_) on the secretion of insulin via the IP_3_ pathway. Through the PKC-dependent signaling pathway, the activation of MT_1_ and MT_2_ heterodimers ultimately causes Ca^2+^ to traverse the plasma membrane, changing the level of insulin excreted. DAG: diacylglycerol; IP_3_: inositol-1,4,5-trisphosphate; PKC: protein kinase C; PLC: phospholipase C. PLC catalyzes PIP_2_ transformation to DAG and IP_3_.

Nevertheless, regardless of the impact on β-cells, the following pathway is clearly involved [31]: PLC is critical for transforming PIP_2_ to IP_3_ and DAG. When IP_3_ binds to the ER, it causes the efflux of Ca^2+^ from the ER to the cytosol [35]. Under the combined stimulatory effects of Ca^2+^, DAG, and phosphatidylserine, PKC is activated, and after successive phosphorylation and activation of various proteins, the transmembrane Ca^2+^ channel opens, enabling entry of Ca^2+^ into the cell. Ca^2+^ influx supplements the endogenous Ca^2+^ levels, and together, these Ca^2+^ pools converge to activate PKC in turn. Hence, a PKC–Ca^2+^ circle forms and endures, through which Ca^2+^ facilitates insulin secretion (Figure 3). Although the impact of the Gα_q_ subunit on PLC remains unclear, increasing evidence tends to support a positive effect. Therefore, many researchers have attempted to elucidate the comprehensive mechanism underlying both the cAMP and IP_3_ pathways to explain the overall inhibitory effect of melatonin on insulin secretion considering the premises of the prevailing theory.

Choosing a placebo as a control and 70% fructose as a common solution for the oral glucose tolerance test (OGTT), Patricia et al. [36] found that the insulin level assayed in the morning declines 30 min after the standard OGTT test. However, when it was assayed in the evening, the insulin concentration declined 90 min after the OGTT test. To understand this phenomenon, they analyzed the difference in blood glucose levels and insulin sensitivity indices in both the morning and evening. They concluded that in the evening melatonin impaired glucose tolerance mainly by decreasing insulin sensitivity and insufficient β-cell compensation, but in the morning, it is mainly through melatonin’s direct inhibition of insulin release. At night, the higher levels of insulin resistance led to a greater demand for insulin, which was needed to metabolize large amounts of blood glucose. Therefore, when compared with its effect on the glucose response in the morning, melatonin will elevate plasma concentrations of insulin to a higher peak more slowly at night.

Their results also showed that melatonin exerts a relatively more significant effect on insulin levels in the evening, with insulin levels increasing at first and decreasing later. To explain this phenomenon, several scientists have provided explanations [34,37,38]. In rat cells stimulated with melatonin, the IP_3_ concentration quickly reached the peak, within the very first minute. Then, it was maintained at an elevated level for at least 15 min and was still at a high level, showing no significant difference compared to the carbachol control group, at 30 min [34]. Although human IP_3_ pathway-mediated insulin secretion has not been elucidated in detail, it has been speculated that human pancreatic cells show IP_3_ level changes similar to those in rodent cells; however, the time point at which the IP_3_ concentration declines may be different [39]. Therefore, insulin levels probably increased through the positive effect of the IP_3_ pathway. Nevertheless, the subsequent decrease in insulin levels could not be explained solely by the presumably positive effect of the IP_3_ pathway. Therefore, researchers reanalyzed the cAMP pathway, integrating it with the IP_3_ pathway to construct a comprehensive signaling system. Many studies [31,37,38] have confirmed that the inhibitory effect of melatonin on the cAMP signaling pathway is realized in approximately 6~8 h. This is much longer than the time required for the activation of the IP_3_ pathway [37,38,40,41]. Hence, the results from the different researchers’ experiments in which INS1 cells or pancreatic cells were used were compared, and it was concluded that the initial increase in the insulin level was probably caused by the rapidly increasing IP_3_ level; however, after a relatively long period, when the inhibitory effect of melatonin was predominantly mediated through the cAMP signaling pathway, the K^+^_ATP_ channel opened, and the L-type Ca^2+^ channel was inhibited, which led to attenuated Ca^2+^ influx, and ultimately, inhibited insulin secretion.

Briefly, the melatonin-induced increase in insulin concentration occurs through the melatonin IP_3_ signaling pathway to compensate for the increasingly fatal insulin resistance at night in humans. This trend ends when the simultaneously activated melatonin cAMP signaling pathway exhibits a higher effect on insulin secretion than the IP_3_ pathway, inducing a decrease in the insulin level. Additionally, the diminishing insulin level is attributed to the gradually reduced stimulatory effect of the decreasing blood glucose concentration, which further drives insulin levels back to normal.

### 3.4. Transcription Factor Controlling Pathways

In addition to the three aforementioned signaling pathways, melatonin can also exert an inhibitory impact on insulin secretion at the genetic level. However, few studies have used human cells to explore the regulatory effect of melatonin on insulin gene expression. The majority of the studies performed to date have been carried out with animals, such as rats, serving as proxies for humans, due to their similar expression of insulin-encoding genes.

The insulin-encoding gene in humans is *INS*, while in rats, it comprises *Ins1* and *Ins2*. In contrast to the majority of vertebrates, rats are exclusively equipped with two independent insulin-encoding genes, *Ins1* and *Ins2*. The evolutionary explanation is based on the complementary functions of two insulin-encoding regions that have enabled adaptation to different environments [42]. In other words, without the simultaneous expression of rat *Ins1* and *Ins2* genes, insulin could not be synthesized in rats, in contrast to humans, in which a single human *INS* encodes insulin. Despite a lack of evidence confirming the tight association between *INS* and *Ins1*, reports have suggested that the *INS* and *Ins2* structures are very similar, with three exons separated by two introns [42]. To confirm the similar function of *INS* and *Ins2*, Karaca et al. [43] were the first to integrate the human *INS* gene into *Ins1/Ins2* double-knockout rats, and they found low insulin production in the transgenic groups. Although the transgenic group expressed only one-half the amount of insulin transcript as the wild-type group, the group concluded that the human *INS* gene and the rat *Ins2* gene resemble each other in structure and transcriptional function. The difference in their expression levels might have been partially attributable to the expression of the *Ins1* gene. Hence, to model *INS*, the study of only the regulatory mechanism underlying *Ins2* expression is insufficient. In the following section, we elucidate the concrete signaling pathways involved from melatonin production to insulin gene (*Ins1*, *Ins2*) expression.

**Figure 4:**
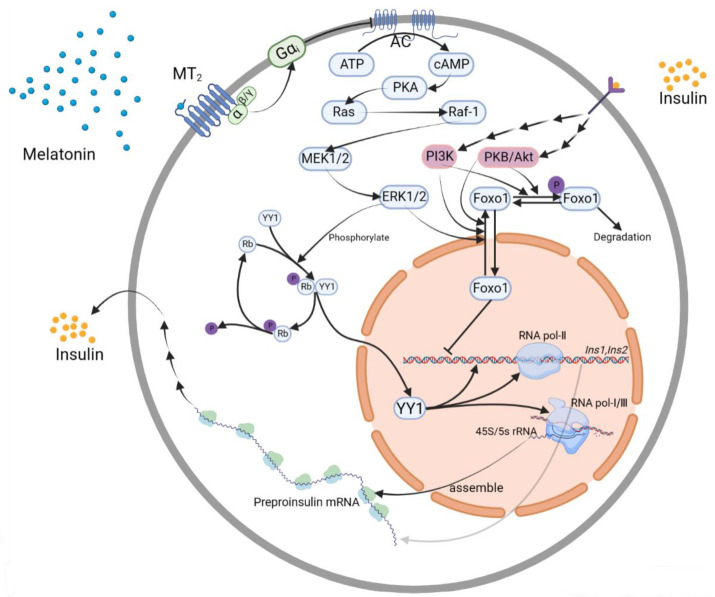
The regulation of the melatonin receptor (MT_2_) to change insulin secretion via insulin gene transcription pathways. The binding of melatonin to MT_2_ activates the downstream MAPK signaling pathway, which results in ERK1/2 activation. Then, through its interaction with YY1 and Foxo1 separately, ERK1/2 participates in the regulation of pre-proinsulin transcription and translation. Insulin also plays a positive role in its own secretion via the PI3K and PKB/Akt signaling pathways. ERK: extracellular signal-regulated kinase; Foxo1: a member of the large family of forkhead rhabdomyosarcoma transcription factors (FKHRs) [53]; MEK: mitogen-activated protein kinase; PI3K: phosphatidylinositol-3-kinase; PKB/Akt: protein kinase B; Rb: retinoblastoma protein; YY1: Yin Yang 1.

Using rats as the experimental subjects, Li et al. [44] discovered that the MAPK pathway played an important role in the effect of melatonin on *Ins1* and *Ins2* expression. The specific signaling process is shown in Figure 4. Through the cAMP-activating process, melatonin activates PKA, which phosphorylates Ras. Then, Raf-1 and MEK1/2 are sequentially activated, and MEK1/2 activates ERK1/2. Finally, ERK1/2 enters the nucleus and promotes the transcription of the *Ins1* and *Ins2* genes. As studies on the receptor MT_1_ are lacking, the study group linked only the inhibitory effect of the receptor MT_2_ to *Ins1* and *Ins2* gene expression via the Ras/Raf/MEK/ERK pathway, suggesting its regulation was mediated through either transcriptional or translational processes. To describe a more detailed mechanism, we thoroughly reviewed the studies focusing on the transcription of *Ins1* and *Ins2*, which are downstream of the MAPK pathway, together with the vital transcription factors involved. Through meticulously performed experiments, YY1 (Yin Yang 1) and Foxo1 were found to work downstream of ERK1/2, exerting positive effects on *Ins1* and *Ins2* transcription in pancreatic cells [45,46,47].

#### 3.4.1. YY1

##### YY1→*Ins1*, *Ins2* Gene

To date, YY1 has been found to regulate insulin expression via three mechanisms. Initially, it can bind to enhancer regions in exon 2 of the *Ins1* and *Ins2* genes, promoting the activity of pre-proinsulin transcription [48]. In addition, YY1 is an RNA pol II factor, which indicates that by interacting with RNA polymerase II, YY1 helps to stabilize the enhancer–promoter interaction during transcription initiation. Therefore, as the mediator between an enhancer and the polymerase, the binding of YY1 is destined to facilitate the transcription of *Ins1* and *Ins2*.

In addition to its impact on transcription, YY1 also plays a role in translation. As a main component of translation, pre-proinsulin mRNA remains untranslated after *Ins1* and *Ins2* have been transcribed. However, for eukaryotic rRNAs, when the levels of synthesized 5.8S, 18S, 28S, and their precursor 45S rRNA are changed, the translation or protein expression of insulin is changed. To date, no relevant reports on pancreatic cells have been published. However, Stoeckius et al. [49] discovered that in human vascular smooth muscle cells, increased YY1 levels in the nucleus can stimulate 45S and 5S rRNA synthesis, indicating that YY1 is involved in the pol I/III-catalyzing transcription of 45S and 5S rRNAs, and thus, promotes insulin expression at the translational level.

##### ERK1/2→YY1

The translocation of YY1 has also been found to be associated with ERK1/2 in the Rb-YY1 cycle [49]. In the cytoplasm, YY1 tends to be coupled with Rb, the retinoblastoma protein, forming the Rb-YY1 complex, and the phosphorylation of this complex by ERK1/2 will facilitate its dissociation. As a result, YY1 is activated and recruited to the nucleoplasm, thereby increasing the transcription levels of the *Ins1*, *Ins2*, and rRNA genes. Differently, phosphorylated Rb remains in the cytoplasm, and after dephosphorylation, reenters Rb-YY1 phosphorylation cycle.

#### 3.4.2. Foxo1

In contrast to YY1, Foxo1 has been found to exert a regulatory effect only on the *Ins2* gene [45]. However, at least three upstream signaling pathways have been identified thus far, among which the Ras/Raf/MEK/ERK pathway, PI3K pathway, and PKB/Akt pathway seem to have the closest relationships with melatonin and insulin secretions. Therefore, these three pathways are described herein.

##### ERK1/2→Foxo1→*Ins2* Gene

The nuclear import of Foxo1 contributes to the suppression of *Ins2* expression [45,50]. More specifically, Foxo1 mainly decreases the transcription level of *Ins2* by interacting with a distal promoter [45]. Mezza et al. [47] suggested that ERK1/2 can directly promote Foxo1 export from the nucleus, independent of the insulin signaling pathway. Therefore, ERK1/2 derepresses the inhibitory effect of Foxo1 on *Ins2* expression. Generally, since MT_2_ initially inhibits ERK1/2, the overall MT_2_ signaling pathway exerts a negative effect on *Ins2* expression.

##### Insulin Receptor→PI3K or PKB/Akt→Foxo1→*Ins2* Gene

The aforementioned signaling pathways link melatonin to insulin and exert negative effects on insulin secretion. However, insulin may play a positive role in its own secretion [51,52]. The mechanisms, clearly understood, involve the PI3K pathway and PKB/Akt pathway. Once insulin binds to an insulin receptor, the upstream signaling pathway is activated, and when the signal is transduced to PI3K and PKB/Akt, both kinases promote Foxo1 export from the nucleus, and then phosphorylate it, facilitating its degradation. Thus, the inhibitory effect of Foxo1 on *Ins2* is derepressed, resulting in enhanced insulin expression. This mechanism is thought to attenuate the negative effect of melatonin on insulin (Figure 4).

## 4. Regulation of *MTNR1B* Expression

### 4.1. Sunshine→MTNR1B Gene Polymorphisms

Since the secretion of melatonin is closely associated with light exposure [11], scientists have questioned whether the expression of melatonin receptors is also regulated by environmental circadian rhythms. Thus, *MTNR1A* and *MTNR1B*, encoding MT_1_ and MT_2_, respectively, have been extensively studied in vertebrates [54,55]. Mainstream genetic research on glucose metabolism or diabetes [12,56] has focused largely on *MTNR1B*, with its higher expression levels and stronger genetic susceptibility association. Therefore, we subsequently focused on *MTNR1B* and MT_2_ expression.

Of all the melatonin pathway-associated disease susceptibility-related single-nucleotide polymorphisms (SNPs), we found that the *MTNR1B* rs4753426C allele frequency was the only one inversely associated with sunshine duration worldwide, indicating that it has largely undergone natural selection on the basis of sunshine duration [13]. Functionally, rs4753426 is located in the promoter region of *MTNR1B* [14], suggesting that it plays a regulatory role in gene transcription. Additionally, this SNP has also been suggested to be significantly associated with T2DM [13]. Moreover, another important *MTNR1B* SNP, rs10830963, has been extensively studied because it is associated with glucose metabolism traits [56,57,58]. Previous studies [59] have demonstrated that the rs4753426C allele and rs10830963G allele are closely linked within the *MTNR1B* gene (*D’* ≥ 0.87, data from the 1000 Genomes Project, Table S1). Therefore, it has been hypothesized that physiological circadian rhythm entrained by environmental sunshine duration probably regulates the expression of melatonin receptor MT_2_ via its direct or indirect control of rs10830963 and/or rs4753426.

### 4.2. MTNR1B Gene→MT_2_ Expression

**Figure 5:**
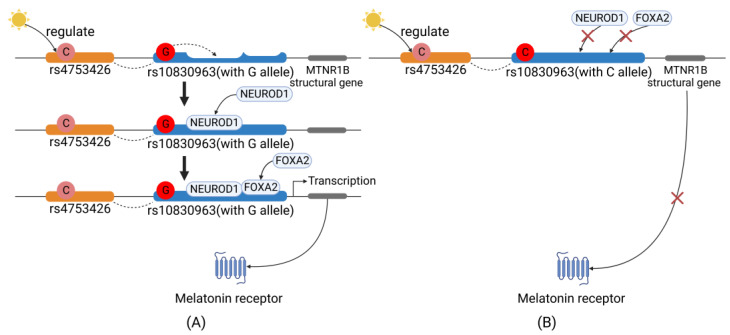
Molecular regulation of sunshine duration on the transcription of the *MTNR1B* gene. Specifically, rs10830963 allelic polymorphism show different MT_2_ expression patterns in response to sunshine duration. (**A**) rs10830963G allele-containing *MTNR1B* and (**B**) rs10830963C allele-containing *MTNR1B*. The dash line indicates linkage disequilibrium between rs4753426C and rs10830963G.

Sunshine duration regulates the activity of the SCN, the key area controlling the central circadian rhythm, specifically the peripheral circadian rhythm clock activity [11]. The SCN releases endocrine hormones to mediate the transcription of *MTNR1B*. Studies have revealed that the rs10830963G allele greatly enhances the expression of MT_2_ [58,60,61,62], with NEUROD1 and FOXA2 [22,63] serving as two important transcription factors in this process. More specifically, as shown in Figure 5, the G allele is carried within a recognition motif in the DNA that matches the consensus sequence of NEUROD1, facilitating NEUROD1 binding to the target site in *MTNR1B* [64]. In addition, the G allele increases the activity of the FOXA2 binding region [64]. Thus, FOXA2 binds to its cis-acting element and interacts with NEUROD1; through their interaction, the bonds between the DNA and both transcription factors are strengthened. Ultimately, the two transcription factors facilitate the transcription of *MTNR1B*, contributing to the higher expression of MT_2_ and lower plasma insulin levels. However, when the G allele is substituted with C, the two transcription factors cannot bind, and MT_2_ transcription is inhibited, which ultimately leads to lower MT_2_ levels, and thus, higher insulin levels in plasma [65].

## 5. Evolutionary Mechanisms

The connection between “low birth weight and high risk of diabetes” was proposed by the thrifty gene hypothesis [66,67], which can also help explain the evolutionary scenario of the *MTNR1B* gene. According to the out-of-Africa theory, humans originated in Africa approximately 200,000 years ago, and adapted behaviorally and genetically to the local sunshine duration [68]. As they were mainly derived from African ancestors, when ancient people migrated to other continents approximately 50,000~100,000 years ago [69], they may also exchange genes with other ancient non-African human populations [70]. After this migration and gene mixture, the original optimum *MTNR1B* gene polymorphisms were subjected to a new round of environmental selection. That is, different sunshine durations determined by locations, as well as different climates, induced novel specific advantageous alleles of *MTNR1B* to accumulate. Then, after a sufficient period of evolution, populations residing in different places demonstrated the alleles in *MTNR1B* at different frequencies, as shown by the significant inverse correlation between sunshine duration and rs4753426C allele frequency [13], and rs10830963G allele frequency.

Relatively recently, the farming society gradually turned into modern society, creating new selection scenarios. In the farming society, people live to a strict rhythm, working at sunrise and resting at sunset, in order to work and harvest more in a limited time. This pattern of life influenced their genes, which were selected such that the expression of the endocrine hormones and their receptors corresponded to the needs established by the outside environment. On the contrary, modern people often must change their natural life rhythms, especially by shortening their sleep time to spend more time with their families and in businesses; in general, they go to sleep late and get up early. According to a report from Sleep Cycle (https://www.sleepcycle.com, accessed on 2 December 2022), which investigated more than 0.94 million people from 40 countries, the daily sleep time for Chinese people has declined to 6 h 43 min (from 0:59 to 7:42). Moreover, the time at which Chinese people went to sleep in 2021, was 1.5 h later than in 2012. People might stay awake longer than what is considered ideal (7–9 h of sleep required [71]), directly prolonging their light exposure. Hence, these people may display low levels of melatonin [72,73,74], but a normal expression level of melatonin receptors owing to unchanged *MTNR1B* expression. In these cases, genes and the environment appear to be maladjusted.

This hypothesis is strongly supported by a meta-analysis, through which 17 cohort studies with multiethnic participants were analyzed. The results indicated that short sleep duration significantly increased the risk of T2DM when compared to that of people who slept for a normal duration (risk ratio (RR) = 1.22, *p* < 0.00001) [75]. Indeed, diminished melatonin secretion was observed in the elderly group in the cohort, who usually slept less on average and for whom the inhibitory effect of melatonin on insulin secretion attenuated. Therefore, these participants displayed elevated insulin levels, representing a greater risk of developing insulin resistance [76], and ultimately, an increase in susceptibility to T2DM. Furthermore, the effect may be pronounced in people eating extra food late at night or eating breakfast early in the morning. Since the insulin levels are not as high as that in the daytime, blood glucose spikes and cannot be absorbed promptly or completely, leading to hyperglycemia and resultant increasing overload of Langerhans’ islets. Accompanied by gradually exacerbating insulin resistance, the untimely rise in blood glucose together with the functionally impaired Langerhans island further contribute to burden the glucose metabolism processes. As a result, these people show a higher possibility of suffering from T2DM.

## 6. Summary

Characterized by insulin resistance and insufficient insulin secretion, T2DM has become a serious global health burden. Except for the regular risk factors, such as obesity and feeding habits identified by most diabetes research, increasing studies have pointed out that shift work and sleep disorders could also elevate the risk of T2DM, suggesting a physiological mechanism of the circadian disruption involved. However, the underlying molecular and evolutionary mechanism linking circadian disruption and T2DM remains to be elucidated. In this review, we initially summarized four major melatonin-stimulated signaling pathways (the cAMP, cGMP, IP_3_, and transcription factor-controlled pathways), focusing on their effects on the regulatory mechanisms of insulin secretion. Then, as exemplified by *MTNR1B*, an evolutionary maladjustment between ancient genes and the present environment was shown to result in circadian rhythm disruption and the exacerbation of impaired glucose metabolism, and even T2DM. Therefore, circadian disruption can be considered a previously unidentified risk factor for T2DM, and the pathological mechanism of this risk was elucidated. This review provides new insights useful to the future prevention, intervention, and treatment of T2DM.

This review also has several limitations. (1) Some studies cited here used non-human species as their experimental materials. Although these animals share a lot in common with humans for glucose metabolism, the differences among species still exist. (2) The evolutionary mechanisms are mainly derived from research on *MTNR1B*, in future other circadian clock genes, like *REV-ERBα* and *BMAL1* [77,78,79,80], should be further included. (3) This review mainly focused on the association between circadian rhythm disruption and T2DM. However, T2DM also has other noticeable contributing factors like obesity [81] and unhealthy dietary patterns [82]. Hence, a future comprehensive analysis of how circadian disruption works with other factors to finally cause T2DM should be considered. (4) All mechanisms explicated here focus on T2DM, whether they apply to other types of diabetes, such as T1DM, should be further explored. Therefore, more comprehensive studies should be guaranteed to better elucidate the pathological mechanisms of T2DM, from the angle of circadian disruption.

## Data Availability

Data authentication is not applicable.

## Supplementary Materials

The following supporting information can be downloaded at: https://www.mdpi.com/article/10.3390/nu15061406/s1, Table S1: Linkage disequilibrium statistics (*D’*) between rs10830963 and rs4753426 in *MTNR1B* gene.
