# Supplementary material for: Molecular Mechanisms of the Melatonin Receptor Pathway Linking Circadian Rhythm to Type 2 Diabetes Mellitus
Source: Nutrients. 2023 Mar 15;15(6):1406. doi: 10.3390/nu15061406 (PMC10052080; doi:10.3390/nu15061406)
Supplement: Supplementary file 1 [file nutrients-15-01406-s001.zip › nutrients-2255283-supplementary.pdf]

Table S1. Linkage disequilibrium statistics (*D'*) between rs10830963 and rs4753426 in *MTNR1B* gene

Race in RegionPopulations	Continent	Variant 1	Variant 2	*D'*
African Caribbean in Barbados	Africa	rs10830963	rs4753426	0.999899
Mende in Sierra Leone	Africa	rs10830963	rs4753426	0.999954
African Ancestry in Southwest US	Africa	rs10830963	rs4753426	0.999999
Colombian in Medellin, Colombia	America	rs10830963	rs4753426	0.999983
Puerto Rican in Puerto Rico	America	rs10830963	rs4753426	0.934714
Han Chinese in Bejing, China	East Asia	rs10830963	rs4753426	0.954272
Southern Han Chinese, China	East Asia	rs10830963	rs4753426	0.932351
Chinese Dai in Xishuangbanna, China	East Asia	rs10830963	rs4753426	0.99996
Japanese in Tokyo, Japan	East Asia	rs10830963	rs4753426	0.999987
Kinh in Ho Chi Minh City, Vietnam	East Asia/South Asia	rs10830963	rs4753426	0.999985
Finnish in Finland	Europe	rs10830963	rs4753426	0.879312
Toscani in Italy	Europe	rs10830963	rs4753426	0.99999
British in England and Scotland	Europe	rs10830963	rs4753426	0.999974
Iberian populations in Spain	Europe	rs10830963	rs4753426	0.959197
Bengali in Bangladesh	South Asia	rs10830963	rs4753426	0.910133
Punjabi in Lahore, Pakistan	South Asia	rs10830963	rs4753426	0.875381
